# Punishment of Abortionists

**Published:** 1868

**Authors:** 


### PUNISHMENT OF ABORTIONISTS.

We are glad to see that some of the vampyres of society are likely to get a share of their just dues. The trial of Dr. G. W. Briggs took place at Providence, R. I., a fortnight since. This, we believe, is the first case that has come to trial under the new statute, which imposes a penalty for the committing of an abortion, of imprisonment in the State Prison of from one to seven years. The testimony of the woman showed that the operation was performed by Dr. Briggs at her own request, her lover, to whom she was then engaged, and was afterward married, accompanying her to the practitioner's office, and paying the bill of $25. Two subsequent operations were necessary before the purpose was accomplished. Upon the body of the child, prematurely born, and born alive, were marks made by the instrument used. The man and woman, soon after this time, went to a place in the northern part of Vermont, and endeavored to live in concealment, for the purpose of avoiding being used as witnesses in this case. As inducements to this course, the man testified that $50 had been handed him in the street by an unknown man, and also a note for $300.—The note was signed " G. W. Briggs," but the genuineness of the signature was not proved before the court. The other evidence presented was that of the physician who was summoned at the inquest, that of the one who attended the woman in her sickness, and that of officer Billings, who made the arrest. The jury, after a short absence from the court-room, returned a verdict of " Guilty !"—*Reporter.*

